# Complex Effects of Cytochrome P450 Monooxygenase on Purple Membrane and Bacterioruberin Production in an Extremely Halophilic Archaeon: Genetic, Phenotypic, and Transcriptomic Analyses

**DOI:** 10.3389/fmicb.2018.02563

**Published:** 2018-10-26

**Authors:** Walter J. Müller, Martha S. Smit, Esta van Heerden, Melinda D. Capes, Shiladitya DasSarma

**Affiliations:** ^1^Department of Microbial, Biochemical and Food Biotechnology, University of the Free State, Bloemfontein, South Africa; ^2^Department of Microbiology and Immunology, Institute of Marine and Environmental Technology, University of Maryland, Baltimore, MD, United States

**Keywords:** purple membrane (PM), bacterioruberin, cytochrome P450, halophilic archaea, bacterioopsin

## Abstract

Halophilic archaea are known to produce a diverse array of pigments for phototrophy and photoprotection. The aim of this paper was to determine the role of a *Halobacterium* gene encoding the predicted cytochrome P450 monooxygenase (CYP174A1) in pigment synthesis through a combined genetic, phenotypic, and transcriptomic approach. We report on the observed phenotype changes [increased bacterioruberin levels and the loss of purple membrane (PM)] between the *Halobacterium salinarum* R1 and its *CYP174A1*-deletion mutant. In addition, we report on the whole-genome DNA microarray analysis, which supports the phenotype of PM loss. This work expands our understanding of the *bop*-gene regulon, and its relation to carotenoid biosynthesis, and sheds light on our broader understanding of the role (s) of CYP174A1 in archaeal pigment synthesis. To date, this is the first study in which the physiological role of any cytochrome P450 monooxygenase (CYP450) in extremely halophilic archaea has been reported.

## Introduction

Members of the *Halobacteriaceae* and *Haloferacaceae* families are extremely halophilic archaea that flourish not only in environments saturated with NaCl but also manage to circumvent harsh environmental factors and survive despite constant exposure to UV- and ionizing radiation and fluctuating levels of desiccation (DasSarma et al., 2006, 2012; Gupta et al., 2015; Rodrigo-Baños et al., 2015). These coping mechanisms, coupled with the fact that these organisms can mostly be cultured with great ease in the laboratory, have sparked much interest due to their potential biotechnological uses (Rodrigo-Baños et al., 2015). The *Halobacterium* and *Haloferax* (Cline et al., 1989; Patenge et al., 2000; Bitan-Banin et al., 2003; Hartman et al., 2010) genera are probably the best studied and most genetically tractable of the *Halobacteriaceae* and *Haloferacaceae* families, respectively – most notable of these is *Halobacterium salinarum* (Sumper and Herrmann, 1976;Oesterhelt and Krippahl, 1983; Ng et al., 2000; Peck et al., 2000, 2001; Berquist et al., 2006). An outstanding feature of *H*. *salinarum* and many other members of the *Halobacteriaceae* family is their red pigmentation that can be attributed to bacterioruberin which is the main carotenoid component in these organisms (Rodrigo-Baños et al., 2015). This red pigment protects *H*. *salinarum* against photo damage due to high light intensities and also aids in photoreactivation and cell membrane reinforcement (Dundas and Larsen, 1963; Shahmohammadi et al., 1998). *H*. *salinarum* also uses light to its advantage by utilizing bacteriorhodopsin (BR) as a light-driven proton pump to generate cellular energy. BR is a simple protein-cofactor complex comprising bacterioopsin (BO) protein and a covalently bound all-*trans*-retinal co-factor. Under microaerobic conditions, BR formation is induced (Krebs et al., 1991) and accumulates to high levels to form a two-dimensional crystal known as the purple membrane (PM) (Peck et al., 2001; Dummer et al., 2011).

Cytochrome P450 monooxygenases (CYP450s) are of special interest due to their versatile biocatalytic repertoire: they can perform an array of reactions including hydroxylation, epoxidation, dealkylation and dehalogenation. (McLean et al., 2005; Bernhardt, 2006). Archaeal CYP450s have received less scrutiny than Bacterial and Eukaryal enzymes and the only two well studied archaeal CYP450s to date are from the hyperthermo-acidophiles *Sulfolobus acidocaldarius* and *Sulfolobus tokodaii* (Koo et al., 2000; Yano et al., 2000; Oku et al., 2004) and the thermo-acidophile *Picrophilus torridus* (Futterer et al., 2004; Ho et al., 2008). Due to the genetic tractability and ease of culturing, we investigated the role of CYP450s in extremely halophilic archaea using the model archaeaon *H. salinarum*. A simple DELTA-BLAST, using the CYP174A1 amino acid sequence from *H. salinarum* R1 as query against the non-redundant database, produced positive hits of more than 460 putative CYP450s spanning 34 genera in total from both the *Halobacteriaceae* and *Haloferacaceae* families. Surprisingly there is no literature available on this specific CYP450 in *H. salinarum* or any other CYP450s from extremely halophilic archaea for that matter.

Currently the physiological function of *CYP174A1* in *H. salinarum* and CYP450s in other extremely halophilic archaea is unknown. Our results provide a possible first clue: the *CYP174A1* from *H*. *salinarum* appears to play a role in pigment metabolism. The role of CYP450s in pigment metabolism is not a novel one and has been well documented in the cheese ripening bacterium *Brevibacterium lines* (Dufossé and de Echanove, 2005), the green algae *Haematococcus pluvialis* (Schoefs et al., 2001), the thermophilic, yellow-pigmented bacterium *Thermus thermophilus* HB27 and the heterobasidiomycetous yeast *Xanthophyllomyces dendrorhous*. In the two aforementioned organisms, their CYP450 encodes for a β-carotene hydroxylase (Blasco et al., 2004; Mandai et al., 2009) and astaxanthin synthase (Ojima et al., 2006; Barredo et al., 2017) respectively.

In this paper we present the very first data that could provide a clue in elucidating the role of CYP450 in the *Halobacterium* genus and specifically *H. salinarum*. The sole and putative CYP450 gene from *H. salinarum* named *CYP174A1*, was deleted from the chromosome of *H*. *salinarum* and the effect of this deletion was evaluated with *inter alia* DNA-microarray analyses.

## Experimental Procedures

### Culturing Conditions

Propagation of pGEM-T^®^ Easy in TOP10 *Escherichia coli* (Invitrogen) was performed in Luria-Bertani (LB) broth (Sambrook et al., 1989) at 37°C with agitation at 160 rpm. Selective pressure was maintained by supplementing the LB broth with ampicillin (final concentration100 μg/mL). Solid media cultivations were performed by supplementing the growth media with 15 g/L bacteriological agar and selective pressure was maintained with 60 μg/mL ampicillin (final concentration).

*Halobacterium* *salinarum* R1 (Stoeckenius and Kunau, 1968; Strahl and Greie, 2008; DasSarma et al., 2018) was cultured in complete medium described by Oesterhelt and Krippahl (1983) that contained (per 1 L): 20 g MgSO_4_ 7H_2_O, 3 g tri-sodium citrate, 250 g NaCl, 2 g KCl and 10 g peptone. Strains were cultured at 40°C at a shaking speed of 200 rpm. Solid media cultivations were performed by supplementing the broth with 15 g/L bacteriological agar and selective pressure was maintained with a final concentration of 10 μg/mL mevinolin (lovastatin) dissolved in dimethyl sulfoxide (DMSO) (final DMSO concentration in medium was 0.1% v/v). For blue/red selection experiments, plates were spread with 40 μL of 20 mg/mL X-gal (5-bromo-4-chloro-3-indolyl-beta-D-galacto-pyranoside; 40 mg/mL dissolved in dimethyl formamide and diluted with water).

### Deletion Construction

*Halobacterium* *salinarum* R1 (Table 1) was cultured in liquid medium until late stationary phase (OD_600_ = 1.2) and genomic DNA extraction was performed as described by Labuschagne and Albertyn (2007). Sequence specific oligonucleotide primer sets (US_Hind_F and Prom_R) and (Term_F and DS_Bam_R) (Supplementary Table S1) and Expand Long Template Polymerase (Roche Molecular Biochemicals) were used to amplify *ca*. 1kb directly upstream (US) and directly downstream (DS) of the open reading frame of the *CYP174A1* gene. Sub-cloning of the individual US and DS regions and ultimately the US/DS fusion (deletion cassette) were performed in pGEM-T^®^ Easy (Promega). All sub-cloned plasmids were extracted as described by Sambrook et al. (1989) The final US/DS deletion cassette was liberated from pGEM-T^®^ Easy with *Hin*dIII and *Bam*HI endonucleases and directionally cloned into the suicide vector pMKK100 (Koch and Oesterhelt, 2005). The resulting pMKK100 construct was transformed into competent *H*. *salinarum* cells to perform blue/red clone selection (Supplementary Figure S1).

**Table 1:** Strains and plasmids used in this study.

Strain or plasmid	Characteristics	Source (reference)
TOP 10 *Escherichia coli*	Plasmid propagation	Invitrogen
*Halobacterium salinarum* R1	Laboratory strain Gas vesicle deficient	Stoeckenius and Kunau, 1968; Strahl and Greie, 2008
pGEM-T^®^ Easy	Blue/White selection, TA-cloning, Amp^*R*^	Promega
pMKK100	Blue/Red selection, shuttle and suicide vector, bgaH, Amp^*R*^, Mev^*R*^	Koch and Oesterhelt, 2005; del Rosario et al., 2007

Competent *H*. *salinarum* R1 cells were prepared as described by Cline and Doolittle (1987) with some minor modifications as described by Koch and Oesterhelt (2005). After transformation cultures were streaked on plates containing mevinolin and X-gal and incubated at 40°C for 5–7 days or until colonies appeared. Successfully transformed cells *i.e.*, cells harboring the integrated pMKK100 plasmid containing the *bgaH* gene (halophilic β-galactosidase) formed bright blue colonies on X-gal containing plates (Patenge et al., 2000).

When the single colonies became large enough, four blue colonies were picked and transferred to four test tubes containing 5 mL complete medium devoid of mevinolin. Cultures were incubated at 40°C at 100 rpm and after the cultures reached an OD_600_ = 0.3–0.4, they were diluted 200-fold in 35 mL complete medium in 100 mL Erlenmeyer flasks without any mevinolin. Cells were cultured 3 consecutive times to a cell density of OD_600_ = 0.3–0.4 and then finally cultured to a density of OD_600_ = 0.5–0.8 at a shaking speed of 100 rpm. After the final round of culturing, the cell suspensions were diluted 10^−5^ and 10^−6^ fold with complete medium to a final volume of 100 μL. The entire 100 μL was plated out onto complete medium containing X-gal and no mevinolin. Plates were then incubated at 40°C for 5–7 days or until colonies became visible. Plates typically yielded 40–50 colonies and at least 10 red colonies from each plate were picked for the deletion screening experiment and all blue colonies were excluded.

### Δ*CYP174A1* Screening

Red colonies were inoculated in 5 mL complete medium without selective pressure at 40°C until OD_600_ = 0.4. Genomic DNA was extracted as described above. To assess if (i) successful *CYP174A1* deletion and (ii) deletion occurred at the correct locus, two separate PCR reactions were performed. Oligonucleotides (421-F and 424-R) based on gene sequences adjacent to *CYP174A1* were used for the first round of PCR using *Taq* DNA Polymerase (New England Biolabs). Clones that displayed the correct deletion genotype were then subjected to a second round of PCR using locus specific oligonucleotide primers (Int-F and DS-Bam-R) (Supplementary Table S1) and Expand Long Template polymerase (Roche Molecular Biochemicals). Clones that displayed the correct amplicon size were then finally designated as Δ*CYP174A1.*

### Pigment Extraction

*Halobacterium* *salinarum* R1 and Δ*CYP174A1* strains were cultured in 35 ml complete medium as described previously. Five milliliter samples were taken during late stationary phase at 86 h (OD_600_ = 1.4) and 96 h (OD_600_ = 1.5) and centrifuged at 17 000 × *g* for 10 min at ambient temperature. Supernatants were removed and the wet weights of the pellets were normalized. Pellets were extracted with ice cold acetone for 1 h at 4°C with agitation. After each extraction, the pellet was collected by centrifugation at 20 000 × *g* and the red supernatant was collected. The extraction was repeated until the pellets appeared white. One milliliter of the supernatant was dried under N_2_-gas and resuspended in 0.3 mL fresh acetone. The concentrated extracted pigments were subjected to a wavelength scan (200–750 nm, 2 nm intervals) in 96-well UV microtitre plates using a SpectraMax M2 (Molecular Devices).

### DNA Microarrays

Strains of *H*. *salinarum* R1 and Δ*CYP174A1* strains were cultured with agitation at 100 rpm in complete medium in triplicate, at 40°C. Samples for total RNA extraction were taken at the logarithmic- and stationary phases of growth which corresponds to OD_600_ of *ca*. 0.3–1.3, respectively (see Supplementary Figure S3). Total RNA extraction, cDNA synthesis, *Cy*3-dCTP (used for parental strain) and *Cy*5-dCTP labeling (used for deletion strain) of the cDNA and computational analyses of results was performed as previously described (Coker et al., 2007; DasSarma et al., 2012). Agilent microarray data were analyzed using GeneSpring software, version 11.5.1 and Agilent Feature Extraction Software, version 10.5.1.1, was used for background subtraction and LOWESS normalization. The extracted RNA from the three parallel cultures for each strain was standardized to 6 μg and pooled to minimize biological noise. Fluorescently labeled cDNA targets from each strain (representing each phase of growth) were combined in a 1:1 ratio and hybridized in duplicate at 65°C for 15 h on a single Agilent slide containing replicated genes from *Halobacterium* sp. NRC-1 (Müller and DasSarma, 2005).

### Membrane Analysis

*Halobacterium* *salinarum* R1 parental and Δ*CYP174A1* strains were cultured in 1 L complete medium at 40°C at 200 rpm until an OD_600_ = 1.2 was reached (late stationary phase). Cells were harvested at 6 000 × *g* for 10 min at 4°C and the resulting pellets were used for purple and red membrane isolation using sucrose cushion gradients essentially as described by DasSarma et al. (2012). Pellets were resuspended in basal salts solution and the resulting cell lysate was transferred into dialysis tubing and dialyzed against 5 L of water at 4°C with three changes. The cell paste was treated with 50 μL DNase (10 μg/μL) and incubated at 37°C for 1 h while shaking at 180 rpm. The DNaseI digested cell lysates were gently layered onto the sucrose gradients and placed in a balanced SW32 Ti rotor and spun at 132 000 × *g* for 17 h at 18°C in a Beckman Coulter Optima^TM^ L-100 Ultracentrifuge.

### Microarray Data Accession Number

Array data were deposited in the GEO database^1^ under series accession number GSE104012.

## Results

### Deletion of *CYP174A1* Appears to Influence Bacterioruberin Synthesis

**FIGURE 1:**
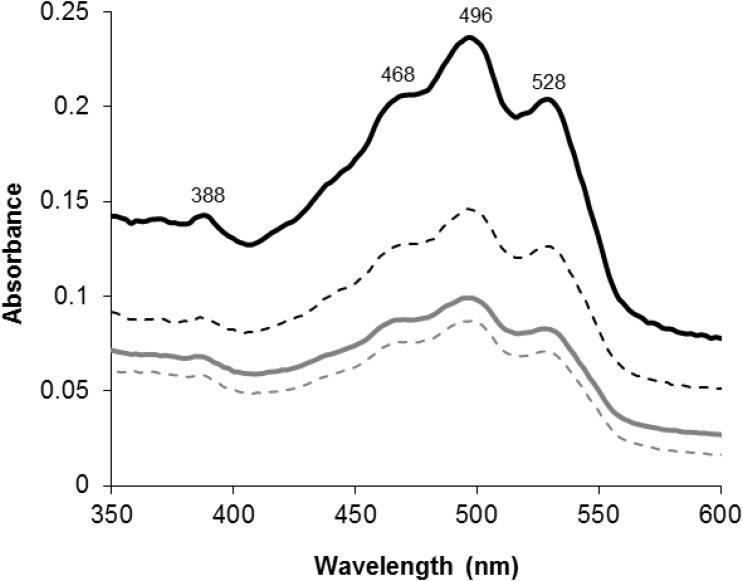
UV-visible spectra of acetone extracted red pigments of cell pellets harvested at 86 h (solid lines) and 96 h (dashed lines) of growth from parental (gray lines) and *CYP174A1*-deletion (black lines) strains of *Halobacterium salinarum* R1. Wavelengths of absorption maxima are indicated on the top scans.

When *H*. *salinarum* R1 parental and the P450 deletion strains were cultured on solid medium and in liquid medium a marked difference in pigmentation was observed. In the liquid cultures the deletion strain first appeared darker red-orange in comparison to the parent R1 strain after about 40 h of growth (early stationary phase). Normalized wet-weight samples of each strain was collected at 86 h and 96 h (late stationary phase) and the red pigments were extracted with acetone and subjected to an UV-visible wavelength scan. Figure 1 illustrates the UV-visible spectra of the extracted pigments from both parental- and deletion strains.

The obtained spectra all displayed the characteristic so-called “three-finger” shape that is typical of the C_50_ bacterioruberin-like carotenoids (D’Souza et al., 1997; Fang et al., 2010). Based on the spectral data, all deletion strains always produced more red pigment when compared to the parental strains. Deletion of *CYP174A1* in *H. salinarum* R1 appears to have influenced carotenoid-metabolism and *inter alia* caused the accumulation of bacterioruberins.

### DNA Microarray and Purple Membrane Analyses

**FIGURE 2:**
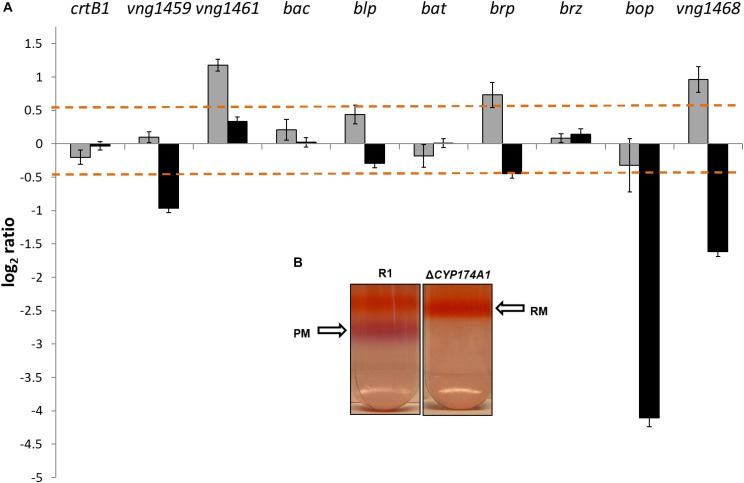
**(A)** Log_2_ ratios (*y*-axis) are shown for expression profiles in logarithmic (gray bars) and stationary growth phases (black bars) for the *H. salinarum* R1 parental and Δ*CYP17*4*A1* strains. Dashed lines indicate the threshold of significant differential expression (see Materials and Methods) and error bars indicate standard errors. **(B)** inlet: *H. salinarum* R1 parental strain and Δ*CYP17*4*A* strain red membrane (RM) and purple membrane (PM) as fractionated by sucrose gradient.

Data from the two color microarrays for samples from the logarithmic and stationary phases of growth were plotted on a scatter plot (Supplementary Figure S2). Differentially expressed genes that were statistically significant were defined as genes that displayed a *P*-value < 0.05 and a fold change cutoff threshold of ≥1.5 (log_2_ ratio of ≥0.5). Based on the aforementioned criteria, 41 and 101 genes were differentially expressed during the logarithmic and stationary phases of growth, respectively. The *brp* gene (encoding the bacterio-opsin-related protein) and two other genes with unknown functions, *vng1461* and *vng1468*, were significantly expressed during the logarithmic growth stage. *Brb*, in conjunction with *crtB1* are involved in the first and last committed steps of the retinal chromophore biosynthetic pathway, respectively (Baliga et al., 2001; Peck et al., 2001; DasSarma et al., 2012). Most strikingly of the gene expression profile, was the very low expression levels (linear fold change of -19.10 or log_2_ ratio of -4.10) of the *bop* gene (encoding for the bacterio-opsin protein) during stationary phase in the Δ*CYP174A1 strain*. The *bop* gene forms part of a cluster of genes, called the *bop*-gene regulon (Peck et al., 2001; Tarasov et al., 2008, 2011; DasSarma et al., 2012) that is involved in the biosynthesis and regulation of BR in PM. Figure 2A illustrates the gene expression profiles of *bop* and other genes associated with the *bop*-regulon during both phases of growth as mentioned above. The very low levels of *bop*-expression imply the possible abolishment of PM synthesis and this was confirmed with a subsequent sucrose gradient: PM was present in the parental R1 strain but absent in the Δ*CYP174A1* strain (Figure 2B).

## Discussion

### Bacterioruberin Accumulation

Deletion of the *CYP174A1* gene in *H. salinarum* R1 appears to have influenced bacterioruberin and PM biosynthesis. Red colored bacterioruberins accumulated (Figure 1) and PM became absent in the Δ*CYP174A1* strain when compared to the parental strain (Figure 2B). An increase in bacterioruberin levels in *H. salinarum* was also observed by Dummer et al. (2011) but this was due a deliberate *bop* gene deletion. The authors discovered that the *lye* (lycopene elongase) gene catalyzed the committed step in bacterioruberin biosynthesis and that the *bop* gene product, bacterio-opsin, inhibited lycopene elongase and consequently the production of bacterioruberins. In the current study, the very low expression of *bop* in the Δ*CYP174A1* strain has likely rendered the lycopene elongase enzyme completely uninhibited and caused the increase in bacterioruberin biosynthesis. It has been previously proposed that free retinal (when not bound to bacterio-opsin) could potentially regulate bacterioruberin biosynthesis; however, Dummer et al. (2011) found that free retinal had no significant effect on bacterioruberin biosynthesis.

### Decreased *Bop* Gene Transcription Levels

The *bop* gene forms part of a tightly regulated cluster of genes referred to as the *bop*-regulon (Tarasov et al., 2008, 2011; DasSarma et al., 2012). *Bop* is regulated by a sensor regulator gene called *bat* and potentially also by a small zinc-finger containing protein called brz. The *bat* gene encodes for a *trans-*acting factor that induces *bop* transcription at low oxygen tension, which naturally occurs in the stationary phase. DasSarma et al. (2012) illustrated that a *bat* deletion caused a marked drop in transcription levels of several genes in the *bop*-regulon including *bop*. The subsequent drop in *bop* transcription directly caused the loss of PM. Figure 2A illustrates that neither *bat* nor *brz* were significantly expressed in stationary phase and that instead of *bop* being induced, the transcript levels of *bop* dropped dramatically, which in turn caused the loss of PM (Figure 2B). In addition, there was no significant gene expression and considerable decrease in transcription levels of e.g., *crtB1* or *blp* as observed when *bat* is deleted (DasSarma et al., 2012). Although a similar phenotype for loss of PM was observed for this study, the crucial difference is that no *bat* deletion was ever introduced as was the case with DasSarma et al. (2012).

In rare cases, the abolishment of *bop* can be attributed to spontaneous insertions either in *bop* itself or in the *brp* gene. Since the early 1980s, several insertion sequences (IS) have been identified in *bop* from various PM-deficient *Halobacterium* strains (Simsek et al., 1982; DasSarma et al., 1983; Ovchinnikov et al., 1984; Pfeiffer et al., 1984; Pfeiffer and Betlach, 1985; Ebert et al., 1987). Typically, the IS called ISH 1 (1 118 bp in size) integrates into a single site in the *bop* gene and ISH 2 (520 bp in size) at several sites of *bop*. In the current study, the *bop*, *brp* and *bat* genes from both the parental and Δ*CYP174A1* strain, were PCR amplified with gene specific oligonucleotide primers (Supplementary Table S1) to assess their ORF size. All the aforementioned genes displayed the correct amplicon size (see Supplementary Figure S4). For the current study, we concluded that IS was most probably not responsible for the decreased *bop* transcription levels and loss of PM in the Δ*CYP174A1* strain.

### Possible Physiological Role of *CYP174A1* in *H. salinarum*

Calo et al. (1995) reported that some species of *Haloarcula hispanica* and *H. salinarum* contain *trans-*astaxanthin. In *H. salinarum* about 11% of the pigment (per weight basis) was *trans-*astaxanthin while 24% was 3-hydoxy-echinenone (Calo et al., 1995). Astaxanthin biosynthesis can occur *via* a 3-hydroxy-echinenone intermediate by the addition of two keto and two hydroxyl moieties at the 4,4′ and 3,3′-positions of the β-ione rings of β-carotene, respectively (Martín et al., 2008). Given the fact that 3-hydroxy-echinenone and astaxanthin have been identified in *H. salinarum* (Calo et al., 1995), we speculate that, as in the case of the CYP450 from *X. dendrorhous*, CYP174A1 from *H. salinarum* acts as both a ketolase and a hydroxylase to produce astaxanthin.

Astaxanthin is a potent anti-oxidant capable of quenching the highly reactive oxygen species (ROS) called singlet oxygen (Makino et al., 2008; Glaeser et al., 2011). Singlet oxygen is the product of photo-oxidative stress due to cells being exposed to high light intensities and can cause severe cell damage by rapidly reacting with *inter alia* proteins, lipids and DNA (Glaeser et al., 2011). Interestingly, microarray analyses by Facciotti et al. (2010) revealed that when *Halobacterium* sp. strain NRC-1 was grown in rich medium, the *CYP174A1* transcript levels significantly increased in the transition from the exponential to stationary phase of growth. Stationary phase is also the period when *bop* expression is increased to biosynthesize PM for the purpose of phototrophic growth (Baliga et al., 2001; DasSarma et al., 2012).

**FIGURE 3:**
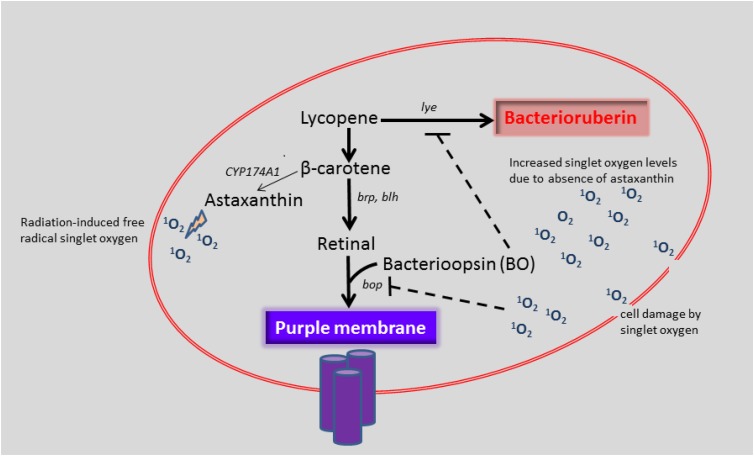
Proposed function of *CYP174A1* in astaxanthin biosynthesis and the effect of increased singlet oxygen levels on *bop* expression in *H. salinarum*. The role of CYP174A1 in astaxanthin biosynthesis in *H. salinarum* has to be verified.

If we assume that CYP174A1 catalyzes the biosynthesis of astaxanthin in *H. salinarum*, it could be argued that increasing levels of singlet oxygen induces the formation of astaxanthin. Interestingly, Schroeder and Johnson (1995) observed that carotenoid biosynthesis was induced by singlet oxygen and other peroxyl radicals in *X. dendrorhous.* The deletion of *CYP174A1* and presumably the consequent decrease in astaxanthin will cause a potential detrimental increase in singlet oxygen levels for *H. salinarum* (Figure 3).

We speculate that when *CYP174A1* is deleted, that increasing singlet oxygen levels and possibly other ROS act as a chemical signal that necessitates *H. salinarum* to utilize an auxiliary mechanism: by inhibiting the *bop* gene product and thereby lifting the inhibition on the *lye* gene. This in turn will result in increased biosynthesis of bacterioruberin, which also has anti-oxidant properties (Rodrigo-Baños et al., 2015). The higher levels of bacterioruberin could further potentially aid in the protection of *H. salinarum* against oxidative damage from singlet oxygen and other ROS.

The role(s) of CYP450s in extremely halophilic archaea is still unknown. This study paves the way for future work that could shed more light on the complex physiological role of CYP450s in not only *H*. *salinarum* but possibly other extremely halophilic archaea. In particular, astaxanthin levels need to be measured in both the Δ*CYP174A1* and parental strains and the possible role of CYP1741 in the synthesis of astaxanthin needs to be verified. The complex regulation of pigment synthesis is not only of interest from a molecular genetic perspective but is also of increasing interest for astrobiology (Schwieterman et al., 2018; Walker et al., 2018).

## Author Contributions

WM performed the research and wrote the paper. MC assisted with the microarray experiments and analysis. MS, EvH, and SD coordinated the study. MS and EvH provided funding for the study.

## Conflict of Interest Statement

The authors declare that the research was conducted in the absence of any commercial or financial relationships that could be construed as a potential conflict of interest.

## Footnotes

1 http://www.ncbi.nlm.nih.gov/geo

## References

[B1] Baliga N. S. Kennedy S. P. Ng W. V. Hood L. DasSarma S. (2001). Genomic and genetic dissection of an archaeal regulon. *Proc. Natl. Acad. Sci. U.S.A.* 98 2521–2525. 10.1073/pnas.051632498 11226271 PMC30170

[B2] Barredo J. L. García-Estrada C. Kosalkova K. Barreiro C. (2017). Biosynthesis of astaxanthin as a main carotenoid in the heterobasidiomycetous yeast *Xanthophyllomyces dendrorhous*. *J. Fungi* 3 1–17. 10.3390/jof3030044 29371561 PMC5715937

[B3] Bernhardt R. (2006). Cytochrome P450 as versatile biocatalysts. *J. Biotech.* 124 128–145. 10.1016/j.jbiotec.2006.01.026 16516322

[B4] Berquist B. R. Müller J. A. DasSarma S. (2006). Genetic systems for halophilic Archaea. *Methods Microbiol.* 35 637–668. 10.1016/S0580-9517(08)70030-8

[B5] Bitan-Banin G. Ortenberg R. Mevarech M. (2003). Development of a gene knockout system for the halophilic archaeon *Haloferax volcanii* by use of the pyrE gene. *J. Bacteriol.* 185 772–778. 10.1128/JB.185.3.772-778.2003 12533452 PMC142808

[B6] Blasco F. Kauffmann I. Schmid R. D. (2004). CYP175A1 from *Thermus thermophilus* HB27, the first β-carotene hydroxylase of the P450 superfamily. *Appl. Microbiol. Biotechnol.* 64 671–674. 10.1007/s00253-003-1529-7 14727092

[B7] Calo P. de Miguel T. Sieiro C. Velazquez J. B. Villa T. G. (1995). Ketocarotenoids in halobacteria: 3-hydroxy-echinenone and trans-astaxanthin. *J. Appl. Microbiol.* 79 282–285. 10.1111/j.1365-2672.1995.tb03138.x

[B8] Cline S. W. Doolittle W. F. (1987). Efficient transfection of the archaebacterium *Halobacterium halobium*. *J. Bacteriol.* 169 1341–1344. 10.1128/jb.169.3.1341-1344.1987 3818549 PMC211943

[B9] Cline S. W. Schalkwyk L. C. Doolittle W. F. (1989). Transformation of the archaebacterium *Halobacterium volcanii* with genomic DNA. *J. Bacteriol.* 171 4987–4991. 10.1128/jb.171.9.4987-4991.1989 2768194 PMC210307

[B10] Coker J. A. DasSarma P. Kumar J. Müller J. A. DasSarma S. (2007). Transcriptional profiling of the model archaeon *Halobacterium* sp. NRC-1: responses to changes in salinity and temperature. *Saline Syst.* 3 1–17. 10.1186/1746-1448-3-6 17651475 PMC1971269

[B11] DasSarma P. Capes M. D. DasSarma S. Das S. Dash H. (2018). “Comparative genomics of halobacterium strains from diverse locations,” in *Microbial Diversity in the Genomic Era*, eds Das S. Dash H. R. (Cambridge, MA: Academic Press), 285–322.

[B12] DasSarma P. Zamora R. C. Müller J. A. DasSarma S. (2012). Genome-wide responses of the model archeaon *Halobacterium* sp. strain NRC-1 to oxygen limitation. *J. Bacteriol.* 194 5530–5537. 10.1128/JB.01153-12 22865851 PMC3458682

[B13] DasSarma S. Berquist B. R. Coker J. A. DasSarma P. Müller J. A. (2006). Post-genomics of the model haloarchaeon *Halobacterium* sp. NRC-1. *Saline Syst.* 2 1746–1748. 16542428 10.1186/1746-1448-2-3 PMC1447603

[B14] DasSarma S. Rajbhandary U. L. Khorana H. G. (1983). High-frequency spontaneous mutation in the bacterio-opsin gene in *Halobacterium halobium* is mediated by transposable elements. *Proc. Natl. Acad. Sci. U.S.A.* 80 2201–2205. 10.1073/pnas.80.8.2201 6300900 PMC393786

[B15] del Rosario R. C. Staudinger W. F. Streif S. Pfeiffer F. Mendoza E. Oesterhelt D. (2007). Modelling the CheYD10K,Y100W *Halobacterium salinarum* mutant: sensitivity analysis allows choice of parameter to be modified in the phototaxis model. *IET Syst. Biol.* 1 207–221. 10.1049/iet-syb:20070007 17708428

[B16] D’Souza S. E. Altekar W. D’Souza S. F. (1997). Adaptive response of *Haloferax mediterranei* to low concentrations of NaCl (< 20%) in the growth medium. *Arch. Microbiol.* 168 68–71. 10.1007/s002030050471 9211716

[B17] Dufossé L. de Echanove M. C. (2005). The last step in the biosynthesis of aryl carotenoids in the cheese ripening bacteria *Brevibacterium linens* ATCC9175 (*Brevibacterium aurantiacum* sp. nov.) involves a cytochrome P450-dependent monooxygenase. *Food Res. Int.* 38 967–973. 10.1016/j.foodres.2005.02.017

[B18] Dummer A. M. Bonsall J. C. Cihla J. B. Lawry S. M. Johnson G. C. Peck R. F. (2011). Bacterioopsin-mediated regulation of bacterioruberin biosynthesis in *Halobacterium salinarum*. *J. Bacteriol.* 192 5658–5667. 10.1128/JB.05376-11 21840984 PMC3187228

[B19] Dundas I. D. Larsen H. (1963). A study on the killing by light of photosensitized cells of *Halobacterium salinarum*. *Arch. Mikrobiol.* 46 19–28. 10.1007/BF00406383 14054143

[B20] Ebert K. Hanke C. Delius H. Goebel W. Pfeiffer F. (1987). A new insertion element, ISH26, from *Halobacerium halobium*. *Mol. Gen. Genet.* 206 81–87. 10.1007/BF00326540 2174546

[B21] Facciotti M. T. Pang W. L. Lo F. Whitehead K. Koide T. Masumura K. (2010). Large scale physiological readjustment during growth enables rapid, comprehensive and inexpensive systems analysis. *BMC Syst. Biol.* 4:64. 10.1186/1752-0509-4-64 20470417 PMC2880973

[B22] Fang C.-J. Ku K.-L. Lee M.-H. Su N.-W. (2010). Influence of nutritive factors on C50 carotenoids production by *Haloferax mediterranei* ATCC 33500 with two-stage cultivation. *Bioresour. Technol.* 101 6487–6493. 10.1016/j.biortech.2010.03.044 20362434

[B23] Futterer O. Angelov A. Liesegang H. Gottschalk G. Schleper C. Schepers B. (2004). Genome sequence of *Picrophilus torridus* and its implications for life around pH 0. *Proc. Natl. Acad. Sci. U.S.A.* 101 9091–9096. 10.1073/pnas.0401356101 15184674 PMC428478

[B24] Glaeser J. Nuss A. M. Berghoff B. A. Klug G. (2011). Singlet oxygen stress in microorganisms. *Adv. Microb. Physiol.* 58 141–173. 10.1016/B978-0-12-381043-4.00004-0 21722793

[B25] Gupta R. S. Naushad S. Baker S. (2015). Phylogenomic analyses and molecular signatures for the class *Halobacteria* and its two major clades: a proposal for division of the class *Halobacteria* into an emended order *Halobacteriales* and two new orders, *Haloferacales* ord. nov. and *Natrialbales* ord. nov., containing the novel families *Haloferacaceae* fam. nov. and *Natrialbaceae* fam. nov. *Int. J. Syst. Evol. Microbiol.* 65 1050–1069. 10.1099/ijs.0.070136-0 25428416

[B26] Hartman A. L. Norais C. Badger J. H. Delmas S. Haldenby S. Madupu R. (2010). The complete genome sequence of *Haloferax volcanii* DS2, a model archaeon. *PLoS One* 5:e9605. 10.1371/journal.pone.0009605 20333302 PMC2841640

[B27] Ho W. W. Li H. Nishida C. R. de Montellano P. R. Poulos T. L. (2008). Crystal structure and properties of CYP231A2 from the thermoacidophilic archaeon *Picrophilus torridus*. *Biochemistry* 47 2071–2079. 10.1021/bi702240k 18197710

[B28] Koch M. K. Oesterhelt D. (2005). MpcT is the transducer for membrane potential changes in *Halobacterium salinarum*. *Mol. Microbiol.* 55 1681–1694. 10.1111/j.1365-2958.2005.04516.x 15752193

[B29] Koo L. S. Tschirret-Guth R. A. Straub W. E. Moënne-Loccoz P. Loehr T. M. de Montellano P. R. (2000). The active site of the thermophilic CYP119 from *Sulfolobus solfataricus*. *J. Biol. Chem.* 275 14112–14123. 10.1074/jbc.275.19.14112 10799487

[B30] Krebs M. P. Hauss T. Heyn M. P. RajBhandary U. L. Korana H. G. (1991). Expression of the bacterioopsin gene in *Halobacterium halobium* using a multicopy plasmid. *Proc. Natl. Acad. Sci. U.S.A.* 88 859–863. 10.1073/pnas.88.3.859 1992477 PMC50913

[B31] Labuschagne M. Albertyn J. (2007). Cloning of an epoxide hydrolaseencoding gene from *Rhodotorula mucilaginosa* and functional expression in *Yarrowia lipolytica*. *Yeast* 24 69–78. 10.1002/yea.1437 17173332

[B32] Makino T. Harada H. Ikenaga H. Matsuda S. Takaichi S. Shindo K. (2008). Characterization of cyanobacterial carotenoid ketolase crtW and hydroxylase ctrR by complementation analysis in *Escherichia coli*. *Plant Cell. Physiol.* 49 1867–1878. 10.1093/pcp/pcn169 18987067

[B33] Mandai T. Fujiwara S. Imaoka S. (2009). A novel electron transport system for thermostable CYP175A1 from *Thermus thermophilus* HB27. *FEBS J.* 276 2416–2429. 10.1111/j.1742-4658.2009.06974.x 19348026

[B34] Martín J. F. Gudiňa E. Barredo J. (2008). Conversion of β-carotene into astaxanthin: two separate enzymes or a bifunctional hydroxylase-ketolase protein? *Microb. Cell Fact.* 7 1–10. 10.1186/1475-2859-7-3 18289382 PMC2288588

[B35] McLean K. J. Sabri M. Marshall K. R. Lawson R. J. Lewis D. G. Clift D. (2005). Biodiversity of cytochrome P450 redox systems. *Biochem. Soc. Trans.* 33 796–801. 10.1042/BST0330796 16042601

[B36] Müller J. A. DasSarma S. (2005). Genomic analysis of anaerobic respiration in the Archaeon *Halobacterium* sp. *strain* NRC-1: dimethyl sulfoxide and trimethylamine *N*-Oxide as terminal electron acceptors. *J. Bacteriol.* 187 1659–1667. 10.1128/JB.187.5.1659-1667.2005 15716436 PMC1064022

[B37] Ng W. V. Kennedy S. P. Mahairas C. G. Berquist B. Pan M. Shukla H. D. (2000). Genome sequence of *Halobacterium* species NRC-1. *Proc. Natl. Acad. Sci. U.S.A.* 97 12176–12181. 10.1073/pnas.190337797 11016950 PMC17314

[B38] Oesterhelt D. Krippahl G. (1983). Phototrophic growth of halobacteria and its use for isolation of photosynthetically deficient mutants. *Ann. Microbiol.* 134B, 137–150. 10.1016/S0769-2609(83)80101-X 6638758

[B39] Ojima K. Breitenbach J. Visser H. Setoguchi Y. Tabata K. Hoshino T. (2006). Cloning of the astaxanthin synthase gene from *Xanthopyllomyces dendrorhous* (*Phaffia rhodozyma*) and its assignment as a β-carotene 3-hydroxylase/4-ketolase. *Mol. Genet. Genomics* 275 148–158. 10.1007/s00438-005-0072-x 16416328

[B40] Oku Y. Ohtaki A. Kamitori S. Nakamura N. Yohda M. Ohno H. (2004). Structure and direct electrochemistry of cytochrome P450 form the thermoacidophilic crenarchaeon, *Sulfolobus tokodaii* str. 7. *J. Inorg. Biochem.* 98 1194–1199. 10.1016/j.jinorgbio.2004.05.002 15219985

[B41] Ovchinnikov Y. A. Zozulya S. A. Zaitseva F. M. Gurev S. O. Sverdlov F. D. Krupenko M. A. (1984). The new insertion sequence element of *Halobacterium halobium* localized within the bacterio-opsin gene. *Bioorg. Khim.* 10 560–563.

[B42] Patenge N. Haase A. Bolhuis H. Oesterhelt D. (2000). The gene for a halophilic β-galactosidase (bgaH) of *Haloferax alicantei* as a reporter gene for promoter analyses in *Halobacterium salinarum*. *Mol. Microbiol.* 36 105–113. 10.1046/j.1365-2958.2000.01831.x 10760167

[B43] Peck R. F. DasSarma S. Krebs M. P. (2000). Homologous gene knockout in the archaeon Halobacterium salinarum with ura3 as a counterselectable marker. *Mol. Microbiol.* 35 667–676. 10.1046/j.1365-2958.2000.01739.x 10672188

[B44] Peck R. F. Echavarri-Erasun C. Johnson E. A. Ng V. W. Kenney S. P. Hood L. (2001). brp and blh are required for synthesis of the retinal cofactor of bacteriorhdopsin in *Halobacterium salinarum*. *J. Biol. Chem.* 276 5739–5744. 10.1074/jbc.M009492200 11092896

[B45] Pfeiffer F. Betlach M. (1985). Genome organization in *Halobacterium halobium*: a 70 kb island of more (AT) rich DNA in the chromosome. *Mol. Gen. Genet.* 198 449–455. 10.1007/BF00332938 2989657

[B46] Pfeiffer F. Friedman J. Boyer H. W. Betlach M. (1984). Characterization of insertions affecting the expression of the bacterio-opsin gene in *Halobacterium halobium*. *Nucleic Acids Res.* 12 2489–2497. 10.1093/nar/12.5.2489 6324122 PMC318678

[B47] Rodrigo-Baños M. Garbayo I. Vílchez C. Bonete M. J. Martínez-Espinosa R. M. (2015). Carotenoids from Haloarchaea and their potential in biotechnology. *Mar. Drugs* 13 5508–5532. 10.3390/md13095508 26308012 PMC4584337

[B48] Sambrook J. Fritsch E. F. Maniatis T. (1989). *Molecular Cloning – A Laboratory Manual*, 2nd Edn, ed. North C. (Harbor, NY: Cold Spring Harbor Laboratory).

[B49] Schoefs B. Rmiki N.-E. Rachadi J. Lemoine Y. (2001). Astaxanthin accumulation in *Haematococcus* requires a cytochrome P450 hydroxylase and an active synthesis of fatty acids. *FEBS Lett.* 500 125–128. 10.1016/S0014-5793(01)02596-0 11445069

[B50] Schroeder W. A. Johnson E. A. (1995). Singlet oxygen and peroxyl radicals regulate carotenoid biosynthesis in Phaffia rhodozyma. *J. Biol. Chem.* 270 18374–18379. 10.1074/jbc.270.31.18374 7629161

[B51] Schwieterman E. W. Kiang N. Y. Parenteau M. N. Harman C. E. DasSarma S. Fisher T. M. (2018). Exoplanet biosignatures: a review of remotely detectable signs of life. *Astrobiology* 18 663–708. 10.1089/ast.2017.1729 29727196 PMC6016574

[B52] Shahmohammadi H. R. Asgarani E. Terato H. Saito T. Ohyama Y. Gekko K. (1998). Protective roles of bacterioruberin and intracellular KCl in the resistance of *Halobacterium salinarum* against DNA-damaging agents. *J. Radiat. Res.* 39 251–262. 10.1269/jrr.39.251 10196780

[B53] Simsek M. DasSarma S. RajBhandary U. L. Khorana H. G. (1982). A transposable element from *Halobacterium halobium* which inactivates the bacteriorhopsin gene. *Proc. Natl. Acad. Sci. U.S.A.* 79 7268–7272. 10.1073/pnas.79.23.7268 6296826 PMC347320

[B54] Stoeckenius W. Kunau W. H. (1968). Further characterization of particulate fractions from lysed cell envelopes of *Halobacterium halobium* and isolation of gas vacuole membranes. *J. Cell Biol.* 38 337–357. 10.1083/jcb.38.2.337 5664208 PMC2107487

[B55] Strahl H. Greie J. C. (2008). The extremely halophilic archaeon *Halobacterium salinarum* R1 responds to potassium limitation by expression of the K + -transporting KdpFABC P-type ATPase and by a decrease in intracellular K +. *Extremophiles* 12 741–752. 10.1007/s00792-008-0177-3 18633573

[B56] Sumper M. Herrmann G. (1976). Biosynthesis of purple membrane: control of retinal synthesis by bacterio-opsin. *FEBS Lett.* 71 333–336. 10.1016/0014-5793(76)80964-7 1001450

[B57] Tarasov V. Y. Besir H. Schwaiger R. Klee K. Furtwängler K. Pfeiffer F. (2008). A small protein from the bop-brp intergenic region of *Halobacterium salinarum* contains a zinc finger motif and regulates bop and crtB1 transcription. *Mol. Microbiol.* 67 772–780. 10.1111/j.1365-2958.2007.06081.x 18179416 PMC2253796

[B58] Tarasov V. Y. Schwaiger R. Furtwängler K. Dyall-Smith M. Oesterhelt D. (2011). A small basic protein from the brz-brb operon is involved in regulation of bop transcription in *Halobacterium salinarum*. *BMC Mol. Microbiol.* 12:42. 10.1186/1471-2199-12-42 21929791 PMC3184054

[B59] Walker S. Bains W. Cronin L. DasSarma S. Danielache S. Domagal-Goldman S. (2018). Exoplanet biosignatures: future directions. *Astrobiology* 18 779–824. 10.1089/ast.2017.1738 29938538 PMC6016573

[B60] Yano J. K. Koo L. S. Schuller D. J. Li H. de Montellano P. R. Poulos T. L. (2000). Crystal structure of a thermophilic cytochrome P450 from the archaeon *Sulfolobus solfataricus*. *J. Biol. Chem.* 275 31066–31092. 10.1074/jbc.M004281200 10859321

