# Supplementary material for: Complex Effects of Cytochrome P450 Monooxygenase on Purple Membrane and Bacterioruberin Production in an Extremely Halophilic Archaeon: Genetic, Phenotypic, and Transcriptomic Analyses
Source: Front Microbiol. 2018 Oct 26;9:2563. doi: 10.3389/fmicb.2018.02563 (PMC6212597; doi:10.3389/fmicb.2018.02563)
Supplement: Supplementary file 1 [file Data_Sheet_1.ZIP › Supplementary material/Figure S1 P450 deletion with pMKK100.docx]

Χ

***bga*H**

Mev^R^

**US**

**DS**

Amp**^R^**

**pMKK100**

**US**

**DS**

***CYP174A1***

Genome

Transformation and 1^st^ crossover event

**US**

**DS**

***CYP174A1***

**US**

**DS**

**Mev^R^**

***bga*H**

**Amp^R^**

Inoculate a single blue colony

Culture in complete medium devoid of mevinolin

**US**

**DS**

***CYP174A1***

**US**

**DS**

**Mev^R^**

***bga*H**

**Amp^R^**

**Second cross over: Option A**

**Second cross over: Option B**

2^nd^ crossover event, loss of *inter alia* the *bga*H gene resulting in no more blue coloured colonies on X-gal plates

Two genotype outcomes. Note: all colonies are now red on X-gal plates

**US**

**DS**

***CYP174A1***

**US**

**DS**

**Outcome of A:** Parental genotype

**Outcome of B:** Deletion of *CYP174A1*

**Fig. S1** Summary of the Blue/Red selection experiment to delete *CYP174A1* by utilizing the pMKK100 suicide vector
