# Supplementary material for: Complex Effects of Cytochrome P450 Monooxygenase on Purple Membrane and Bacterioruberin Production in an Extremely Halophilic Archaeon: Genetic, Phenotypic, and Transcriptomic Analyses
Source: Front Microbiol. 2018 Oct 26;9:2563. doi: 10.3389/fmicb.2018.02563 (PMC6212597; doi:10.3389/fmicb.2018.02563)
Supplement: Supplementary file 1 [file Data_Sheet_1.ZIP › Supplementary material/Figure S3 Growth of H. salinarum R1 parental and deletion strains.docx]

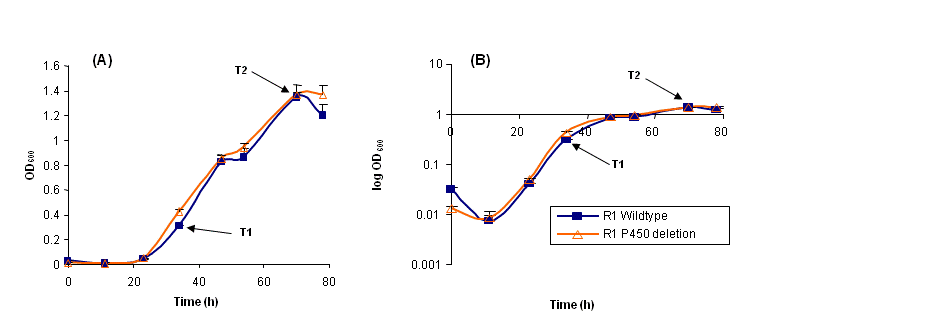


**Fig. S3** Growth of *H*. *salinarum* R1 parental strain (in blue) and Δ*CYP174A1* strain (in orange) respectively at 40˚C. Samples for total RNA extraction were taken at Time 1 **(T1)** and Time 2 **(T2)** which corresponds to the late logarithmic and stationary phases of growth respectively. Error bars are representative of triplicate experiments.
