# Supplementary material for: Complex Effects of Cytochrome P450 Monooxygenase on Purple Membrane and Bacterioruberin Production in an Extremely Halophilic Archaeon: Genetic, Phenotypic, and Transcriptomic Analyses
Source: Front Microbiol. 2018 Oct 26;9:2563. doi: 10.3389/fmicb.2018.02563 (PMC6212597; doi:10.3389/fmicb.2018.02563)
Supplement: Supplementary file 1 [file Data_Sheet_1.ZIP › Supplementary material/Figure S4 PCR amplification of the bat, brp and bop genes.docx]

**2 000 bp**

**1 000 bp**

**500 bp**


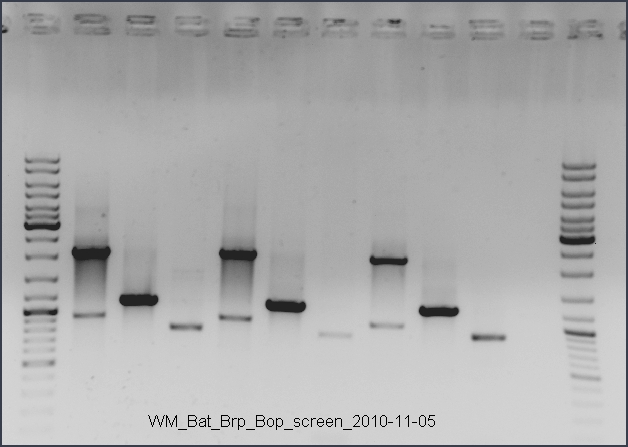


**GR 1 2 3 1 2 3 1 2 3 Neg GR**

**R1 parental**

**∆*CYP174A1***

**NRC-1 wildtype**

**Fig. S4** PCR amplification of the *bat*, *brp* and *bop* genes to screen for the presence of spontaneous insertions. Expected sizes of amplicons in parental as well as the deletion strains of *H*. *salinarum* R1: *bat* = 2 022 bp, *brp* = 1 104 bp, *bop* = 786 bp. For *Halobacterium* sp. NRC-1: *bat* = 2 025 bp, *brp* = 1 080 bp, *bop* = 789 bp. Lanes: **GR** = 5 µL GeneRuler (Fermentas); **1** = *bat*; **2** = *brp* and **3** = *bop* and **Neg** = Negative control.
