# Supplementary material for: Complex Effects of Cytochrome P450 Monooxygenase on Purple Membrane and Bacterioruberin Production in an Extremely Halophilic Archaeon: Genetic, Phenotypic, and Transcriptomic Analyses
Source: Front Microbiol. 2018 Oct 26;9:2563. doi: 10.3389/fmicb.2018.02563 (PMC6212597; doi:10.3389/fmicb.2018.02563)
Supplement: Supplementary file 1 [file Data_Sheet_1.ZIP › Supplementary material/Table S1 Oligonucleotide primers used in this study.docx]

**Table S.1**  Oligonucleotide primers used in this study

| **Oligonucleotide**  **primer name** | **5´ – 3´ DNA sequence** | **Application** | **Restriction sites/ Comments** |
| --- | --- | --- | --- |
|  |  |  |  |
| US_Hind_F | 5'- aag ctt cga agt cgg cgt cct gct c -3' | US-Deletion cassette | *Hin*dIII |
| Prom_R | 5'- ctg cag acg tac gtc tcc atg ggt ccc -3' | US-Deletion cassette | *Pst*I |
| Term_F | 5'- ctg cag aac agg aga tgc gga tgc gg -3' | DS-Deletion cassette | *Pst*I |
| DS_Bam_R | 5'- gga tcc ctg gga cgt cgg cat gag -3' | DS-Deletion cassette | *Bam*HI |
| 421_F | 5'- atg cag gat gcc ggc att cc -3' | P450 deletion screen | Gene specific primers*^a^* |
| 424_R | 5'- tca ctc gtc tac gtg gtc ga -3' | P450 deletion screen | Gene specific primers*^a^* |
| Int_F | 5'- gcg gcc gtt gtg tgg ctg gtt t -3' | Site specific integration | Pair with DS_Bam_R |
| 1464_F | 5'- atg acg agc gtc cag aac acc -3' | *Bat*  gene amplification | Gene specific primers*^a^* |
| 1464_R | 5'- tca ctc ctc gaa gaa cgc tcc -3' | *Bat*  gene amplification | Gene specific primers*^a^* |
| 1465_F | 5'- atg ctc ggt agt gac gtg tgt -3' | *Brp* gene amplification | Gene specific primers*^a^* |
| 1465_R | 5'- tca tgg gac gta cca gat gcc -3' | *Brp* gene amplification | Gene specific primers*^a^* |
| 1467_F | 5'- atg ttg gag tta ttg cca aca -3' | *Bop* gene amplification | Gene specific primers*^a^* |
| 1467_R | 5'- tca gtc gct ggt cgc ggc cgc -3' | *Bop* gene amplification | Gene specific primers*^a^* |

*^a^*Oligonucleotides were a generous gift from Prof. Shiladitya DasSarma, Department of Microbiology and Immunology, University of Maryland (Baltimore), Maryland, USA.
